# Normal Ovariotomy

**Published:** 1874-09

**Authors:** J. T. Gilmore

**Affiliations:** Professor of Surgery in the Medical College of Alabama; Mobile, Ala.


ATLANTA

MEDICAL AND SURGICAL JOURNAL.

VOL. XII.] SEPTEMBER—1874. [No. 6.

### Original Communications.

### NORMAL OVARIOTOMY.

BY J. T. GILMORE, M.D.,
*Professor of Surgery in the Medical College of Alabama.*

During the spring of 1868, I was invited by Dr. Gaines of our city to see with him a Mrs. G., who was suffering from amenorrhœa. She was at that time a beautiful plump young woman of about twenty-three years. Her history was, that at the age of fifteen years she commenced menstruating. Her menses occurred irregularly up to the time of her second marriage, at which time it entirely ceased, having seen nothing since. Her second marriage was two years previous to my seeing her. Her first marriage was in her eighteenth year; her first husband living only three years, she remaining a widow only a year. Her chief source of trouble from the amenorrhœa was her constant headache, of which she complained more or less constantly; yet accompanied by severe paroxysms at the time of each menstrual epoch, recurring every lunar month. A physical examination exhibited her to be a well-formed woman; her mammary glands were perfectly developed; her limbs well rounded; her mons veneris was covered with the usual growth of hair; her voice was possessed of feminine softness.

The speculum could be readily introduced into the vagina without eliciting any complaints of pain. The finger introduced before resorting to the speculum, found a little nodule in the vault of the vagina; the cervix uteri not exceeding in length one-quarter of an inch; and above we recognized the absence of the usual weight that a well-developed uterus imparts to the finger when palpation is resorted to. Placing her in Sims' obstetrical position, and using his speculum, we recognized the cervix, which we steadied with a tenaculum, and without difficulty passed a sound into the cavity of the uterus to the depth of one and a quarter inches.

This cleared up the diagnosis: here was a woman in all probability with ovaries, and without a uterus, or with only the remains or rudiments of a uterus; and we could not hope to restore the function of the organ unless we could stimulate it to grow to something near its normal size, by means mainly of local applications, such as sponge tents, electricity, etc., and in the meantime control, as far as possible, the paroxysms of congestive headache by appropriate means, taking care that the brain should not incur any damage if it were possible to prevent it.

This was the course of treatment Dr. Gaines pursued. She remained in his charge for some two years; and as all such cases do, she applied to nearly all the more prominent physicians, and among them was my friend, Dr. Ketchum, who made out her case to be one of atrophy of the uterus, and followed the same plan of treatment.

When I first saw her with Dr. Gaines, she was nervous and hysterical, yet possessing considerable mental sprightliness. When she applied to me in the fall of 1872, begging that something should be done for the relief of her sufferings, complaining that her headaches were constant; and every fourteen days, without fail, a paroxysm of congestion of the brain would come on, and give rise to convulsions that would last for several days. During the intervals between these convulsions she would suffer from diplopia, and her acuteness of vision was permanently impaired. At the time this patient applied to me, Prof. Battey, now of the Atlanta Medical College, had introduced to the profession his new procedure for the relief of these congestions of the brain that imperil life, due to amenorrhœa, irremediable by any of the already recognized remedies, viz: removal of the ovaries. In her appearance and development there was every evidence of well-developed ovaries. She and her husband both assured me that she possessed the usual amount of sexual appetite. This operation at once occurred to me as a proper expedient in her case; but before suggesting it I consulted with Dr. Ketchum, who was more familiar with her condition than I was, and he expressed the opinion that unless something was speedily done for her relief she could not survive many more attacks. Her physiognomy was surely indicative of some grave brain mischief. Her mental hebetude between the paroxysms was increasing; unsteadiness of gait was also progressive.

In deciding upon the propriety of this operation in any case, the question hinges upon the single point—are the ovaries the source of the menstrual irritation? The successful double ovariotomies have unsettled the professional mind upon this physiological point, which in former times was declared emphatically settled; every writer upon physiology declaring that ovulation was in some way connected with menstruation. The question, is the menstrual flow a secretion, or is it a hemorrhage? has long been a subject of discussion, and still remains undetermined; yet I believe the weight of modern testimony is in favor of its being a secretion. If it is a hemorrhage, some sort of stimulation to bring about a congestion of the pelvic organs, mainly the uterus, is essential; if it is a secretion, the only requisite is an organ competent to discharge the function, controlled by the necessary vitalizing force. For my part, I lean to the latter opinion. I believe the menstrual flux to be a secretion, and the uterus a secreting organ.

If we take up the physiological development of puberty we find many interesting phenomena for study; *pari passu* with the development of the genital organs, commencing at puberty, we note the change of voice, the growth of hair on the pubis, and of the beard in the male. There is an impetus imparted to the nervous system, pervading the entire organization, that marks and makes manifest the differences that exist between childhood, adolescence, manhood, and womanhood. That these changes are due mainly to the development of the genital organs is indisputably true; for unsexing either the male or the female before these changes have taken place prevents them, and the animal becomes a nondescript. But, after the full development of womanhood, it remains to be proven that the removal of the ovaries will rob the nervous system of the impression already made by their growth and vitalizing force. All experience, indeed, seems to negative this idea. With the growth of the ovaries and uterus in the female, and the testicles and penis in the male, with the concomitant changes elsewhere, already mentioned, there is a nervous influence produced in the nerves supplying the genitalia, that remains even after their ablation. Hence it is that a stallion, castrated after he has indulged his sexual propensities, retains to some degree his appetite for the opposite sex. All animals, so far as I know, both male and female, after having matured and indulged their sexual appetites, retain their sexual desires even after being unsexed. Our Creator seems to impart to these organs a nervous influence, at the time of their commencing activity, that impresses the whole organization. We note in them the perfection of development and activity, which in the female commences with the menstrual show. These organs retain the perfection of organization during the menstrual activity, and with its decline undergo senile decay or fatty degenerations, and lose their important connection with the organization; or, to speak more pointedly, the ovaries, uterus, and breasts grow at puberty until womanhood is attained; during the menstrual period until the climacteric period of female life these organs preserve their integrity of organization; then they undergo structural alterations, which, under constitutional vice, oftentimes develop cancer. With this structural alteration there is a decline of the nerve influence; but this nerve influence, when once created, seems to be largely independent of the organs that created it, and lives on its allotted space of time independent of the ovaries. The creation of this impression upon the nerves, supplying the genital organs, is one of nature's most mysterious workings; a full appreciation of which is indispensable to a correct understanding of many of the diseases that belong to puberty and womanhood. Any exaltation or want of proper equilibrium of this genital nerve power, without organic disease, in the female begets hysteria and in the male spermatorrhœa, and in both sexes that anomalous condition called spinal irritation. We must admit that in nearly all cases of hysteria, of spermatorrhœa, and spinal irritation, that there is a marked predisposition, oftentimes a hereditary tendency, and most often do we find this ataxic diathesis in children born of consumptive parents, who early in childhood were the subjects of convulsions; wet the bed at night; and in boys, when puberty commenced, they were troubled with seminal emissions.

It is the derangement of this special sexual life that gives the practitioner his greatest difficulty. It is here more than elsewhere that he encounters the invisible, undemonstrable part of pathology; and it is the derangement of this special nerve force that has given rise to so many of the various surgical procedures: Isaac Baker Brown's clitoridectomy; Burns' and Sims' operations for vaginismus; Nott's and Simpson's operations for cocyodinia; forcible dilatation of the sphincters of the vagina and rectum for the relief of hysterical vesical tenesmus; Lallemand's *porte caustique* and cauterization of the prostatic urethra; over distension by the various appliances; Trousseau's rectum plug, prostatic compressor, and his recommendation of forcible dilation of the anus. All of these procedures, empircally instituted, have for their purpose the toning down of a hyperæsthetic exaltation of that nerve power of the sexual organization that is evolved by the growth and development of the ovaries in the female and the testicles in the male.

Castration will not cure spermatorrhœa. Will ablation of the ovaries cure hysteria? When this special genital nerve life is once created it must live out its natural life, and cease by the limitation that our Creator has given it. Now it is possible that the removal of the ovaries may modify this nerve power, and may in this way become a legitimate surgical resource in the treatment of a certain class of cases. The function of the ovaries may give an impetus to the menstrual molimen when it takes a wrong direction, that begets harm; but this question is still *sub-judice*, and can only be determined by clinical experience. That it cannot be relied upon as a sure remedy to do away with the menstrual flow is well established. Then, in my judgment, normal ovariotomy can only modify that special nerve power that resides in the nerves supplying the female genitalia evolved by the development of the ovaries. And whether it will do this in a manner so as to lessen the dangers that threaten life when an important organ or organs are assailed by a misdirection of the irritation, can only be known by actual experiment.

But to resume the history of Mrs. G.'s case: After talking over the subject with my colleague, Dr. Ketchum, and also Dr. W. H. Anderson, who fills the chair of physiology in the Alabama Medical College, we decided to make the experiment, if she and her husband would consent. The experimental character of the operation, and its dangers, were fully explained to them, and, as most persons in her condition would have done, she and her husband eagerly sought it, and begged that it should be done as early as possible.

At the Sisters' Infirmary, on the 18th day of December, 1872, in presence of Drs. Anderson, Gaines, Owen, Goode and Sawyer, the operation was done, by carefully making an incision into the abdominal cavity, just above the preperitoneal space in the median line, some three inches in length. The abdominal walls were unusually thick, and there was a considerable quantity of fat beneath the posterior layer of the sheath of the recti abdominis muscles. After gaining access to the cavity, two fingers of the right hand were passed into the pelvis, and the right ovary was readily seized and brought out through the abdominal incision. A double ligature of delicate silk was passed beneath the ovary through the broad ligament, and each half of that portion of the broad ligament that supported the ovary was tightly ligated by the silk threads introduced; the ovary was then excised. Search was then made for the left ovary, and it was with some considerable difficulty that it was found. By locating the uterus, which was atrophied, as already described, and agglutinated to a mass of small intestine, (indeed, it was imbedded in the mass of intestine, which was everywhere adherent to it, thus hiding it from our search,) then following the course of the left broad ligament, (upper portion) which was also concealed by adherent intestine, the left ovary was dug out of this mass, by breaking up some of the adhesions, and brought to the surface of the abdomen through the incision, and disposed of as had been done with its fellow of the right side. Deep silver wire sutures were then passed through the entire thickness of the abdominal walls, and the incision was closed.

She had been prepared for the operation by having the bowels well opened; and as soon as she was put to bed a full dose of opium was administered. This was repeated every four or six hours. The succeeding morning she was found with a considerable rise of fever, with an anxious expression of countenance, and some swelling of the abdomen. The opium was liberally pushed, and tinct. veratri. vir. was given also in decided doses, until the fever and peritoneal inflammation were controlled.

On the fifth day the sutures were removed, and the whole abdomen was encircled by broad straps of adhesive plaster. At the end of the second week there was a recurrence of the fever, which subsided in the morning and returned in the evening. By examining the vagina and the lower part of the abdomen, a very considerable swelling was found in the left broad ligament. In despite of treatment, this gradually increased, extending upwards until it reached the lower part of the abdominal incision, which had entirely healed; and at the end of two more weeks it opened spontaneously through the incision at its lowest point, discharging a large quantity of fetid, thin pus. I then, with a gum catheter introduced into the cavity, washed it out twice daily with carbolized tepid water. The abscess was formed in the left broad ligament, which I am quite sure had been, in the earlier days of her menstruation, the site of an attack of cellulitis, and in all probability brought about the atrophy of the uterus by involving possibly the uterine tissues, or in some way interfering with the uterine arteries, thus cutting off the supply of blood to the uterus.

A more unfavorable case for this operation could scarcely have been found. Her nervous system was so completely shattered, both by her disease and the large and repeated doses of calomel and iodide and bromide of potassium, and the drastic purgatives, that had been given to relieve the brain; indeed, at the time of the operation, mercurial fetor was noticeable in her breath; and of course all of these influences favored the formation of an abscess at the point at which it developed, and rendered its prevention almost impossible. The use of the catheter and carbolized water twice a day soon arrested her fever, and with the aid of tonics she commenced regaining her appetite and strength. She was confined to her room in all about two months. When she got up a considerable portion of the incision gave way, and a large ventral hernia formed, for which she wore a truss. The weather soon grew very hot, and our long summer was upon us. The hot weather and constant drain from the abscess rendered rapid improvement impossible; yet she constantly gained ground until I left on the 25th of August on a visit of recreation to Tennessee for two weeks. When I left her the discharge was so small that, not having used the catheter for some time, I did not anticipate any return of a necessity for its employment.

During my absence my friend Dr. Rhett Goode called to see her, and he found her collapsed. Feeling alarmed at her condition, he called in Dr. W. H. Anderson, who pronounced her depression as due to the reabsorption of septic fluid from the abscess, and advised him to probe it, which he did, and re-established the flow, which had entirely ceased previous to the attack.

On my return I found that she had lost much that she had gained, and that she was scarcely able to be out of bed; appetite gone, and suffering more or less daily from hectic. I had her carried back to the infirmary in order to have the use of the catheter resumed. Unfortunately about the time of my return a case of yellow fever occurred in the City Hospital, just opposite the infirmary, and in a few days the influence was brought to the infirmary, killing one of the sisters of charity and my patient.

She had commenced improving under the influence of the treatment, by the use of the catheter and the carbolized tepid water. Had it been possible to open the abscess through the the vagina, her recovery would have been certainly assured. But the abscess had formed in the upper portion of the broad ligament, and had not approached near the vault of the vagina; and the probable adhesions of the intestines to the parts made any attempts to reach it through the vagina extremely hazardous. A solid bougie introduced through the abdominal opening into the abscess, and its point made to reach as far as possible in the direction of the vagina, indicated that it would be unsafe to attempt to reach it through the vagina. Therefore, our only chance of affording relief was by the means already indicated. The constant drain from the abscess, possibly supplementing the menstrual flow, and relieving the molimen, robbed this case of all important bearing on the merits of this operation. The misdirection of the menstrual irritation was certainly corrected either by the discharge from the abscess or the removal of the ovaries. The ovaries, both about the same size, were not more than two-thirds of the natural size; yet, so far as I could judge with the unaided eye, they were healthy. There were some hysterical symptoms remaining after the operation that increased every two weeks; but nothing approaching convulsions was observed. A sense of fullness about the throat, and a feeling as though a hair was titilating the larynx, and accompanied with a free flow of saliva and a general figitiness, were the most prominent hysterical symptoms.

Then, to resume briefly this case: Here was a woman with irregular menstruation from the commencement of her menstrual life, probably caused by an attack of pelvic cellulitis, ending in complete atrophy of the uterus and amenorrhœa, without a corresponding diminution of the menstrual impulse and wasting of the ovaries. She worries through a period of over ten years, with her brain constantly subjected to this irritation, which nature, in her wisdom, intended should have its outlet through the monthly uterine secretions or hemorrhage. Her brain had been protected, as long as it was possible, by purgatives and those sedatives that calm by their physiological action that peculiar nerve force that is a part of the genital organization; and something had to be done, else her brain, already impaired, would give way, and she would find relief only in an early death. Under these circumstances, normal ovariotomy was resorted to.

Since operating upon this case I have met with two other cases of wasted uteri; one of atrophy in a woman, who had in early life menstruated regularly. She is now thirty-nine years of age, and for the past ten years has seen nothing. She married about a year before the amenorrhœa came on. The atrophy in this case undoubtedly followed an attack of cellulitis; for the characteristic stiffness of the broad ligaments and the nodules still remain. The other is a case of hyperinvolution following her third confinement; she is now forty years old. Both of these cases suffer very great inconvenience from the absence of the function; yet their lives are not seriously threatened by the retained molimen assailing either the brain, lung or stomach; and of course in these cases normal ovariotomy is not thought of.

Now, in appreciation of this operation, I can only state, that in my experience I can recall only four cases in which I would have contemplated the removal of the ovaries with the present light before me. In all of these cases there was an atrophy or a congenital want of development of the uterus; and all of them died. Cases of ovaritis, or ovarian irritation, may arise which misdirect the molimen, and so imperil life as to justify their removal; but I have not seen such cases. I do not believe that the removal of the ovaries could in any instance be resorted to with an expectation that the menstrual function would certainly be abolished. Nor do I believe that normal ovariotomy would cure hysteria, any more than ablation of the testicles would cure spermatorrhœa; for, to my mind, the hysteria of early womanhood, when not due to actual congestion or disease of the uterus or ovaries, like spermatorrhæa, is, in the majority of instances, found in persons of a nervous diathesis, who have practiced, to some extent, masturbation, and deranged the equilibrium of the nerve power that is a part of the sexual organization. In these cases I would no more think of normal ovariotomy than I would of castration for spermatorrhœa. Yet, if I was dealing with an ulcer of the stomach, or gastritis, due to the menstrual molimen, fixed in the solar plexus, that could not be dislodged by remedies, and life was seriously jeopardized, I would entertain thoughts of normal ovariotomy. I might say the same in regard to the brain and lungs. I have never yet met with a patient with a well-developed uterus in whom I would have consented to the operation. It is in cases of congenital absence of the uterus, with the menstrual molimen threatening life; also in cases of atrophy, or hyperinvolution of the organ, with life endangered, that I would, relying upon my experience, give the operation a trial. It is true, that in suitable cases the operation is not one of more danger, when it can be practiced through the vagina, than many others that are resorted to for convenience. Yet the unsexing of an individual can only be compensated for by the rescuing of life from certain death; and it is for this purpose only that I would resort to it, fully understanding and explaining that it was experimental, until a sufficient number of cases have been subjected to it to give the operation its proper place as a remedial procedure.

